# Effect of fat contents of buttermilk on fatty acid composition, lipolysis, vitamins and sensory properties of cheddar-type cheese

**DOI:** 10.3389/fmicb.2023.1209509

**Published:** 2023-08-30

**Authors:** Mussab Asif, Muhammad Nadeem, Muhammad Imran, Rahman Ullah, Muhammad Tayyab, Faima Atta Khan, Fahad Al-Asmari, Muhammad Abdul Rahim, João Miguel Rocha, Sameh A. Korma, Tuba Esatbeyoglu

**Affiliations:** ^1^Department of Dairy Technology, University of Veterinary and Animal Sciences, Lahore, Pakistan; ^2^Department of Food Science, Faculty of Life Sciences, Government College University, Faisalabad, Pakistan; ^3^Department of Animal Products Technology, The University of Agriculture, Dera Ismail Khan, Pakistan; ^4^Institute of Biochemistry and Biotechnology, University of Veterinary and Animal Sciences, Lahore, Pakistan; ^5^Department of Food and Nutrition Sciences, College of Agricultural and Food Sciences, King Faisal University, Al Hofuf, Saudi Arabia; ^6^Laboratório Associado, Centro de Biotecnologia e Química Fina (CBQF), Escola Superior de Biotecnologia, Universidade Católica Portuguesa, Porto, Portugal; ^7^LEPABE—Laboratory for Process Engineering, Environment, Biotechnology and Energy, Faculty of Engineering, University of Porto, Porto, Portugal; ^8^ALiCE—Associate Laboratory in Chemical Engineering, Faculty of Engineering, University of Porto, Porto, Portugal; ^9^Department of Food Science, Faculty of Agriculture, Zagazig University, Zagazig, Egypt; ^10^School of Food Science and Engineering, South China University of Technology, Guangzhou, China; ^11^Department of Food Development and Food Quality, Institute of Food Science and Human Nutrition, Gottfried Wilhelm Leibniz University Hannover, Hannover, Germany

**Keywords:** cheddar, cheese, butter, organic acid, food development, lactic acid bacteria

## Abstract

Cheddar-type cheese produced from buttermilk had softer texture than standard cheddar cheese due to lower fat content of buttermilk. Fat is extremely important for the functional characteristics and optimum textural attributes of cheese. The effect of different fat contents of buttermilk on chemical characteristics of cheddar-type cheese is not previously investigated. This investigation was conducted to know the effect of different fat contents of buttermilk on fatty acids composition, organic acids, vitamins, lipolysis and sensory characteristics of cheddar-type cheese. Cheddar-type cheese was produced from buttermilk having 1, 1.75, 2.50 and 3.25% fat contents (control, T_1_, T_2_ and T_3_). Fat content of control, T_1_, T_2_ and T_3_ were 9.81, 16.34, 25.17 and 31.19%. Fatty acids profile was determined on GC–MS, organic acids and vitamin A and E were determined on HPLC. Free fatty acids, peroxide value and cholesterol were determined. Cheddar-style cheese produced from buttermilk (1% fat) showed that it had softer texture and lacking typical cheese flavor. Gas chromatography–mass spectrometry (GC–MS) analysis showed that long-chain unsaturated fatty acids in control, T_1_, T_2_ and T_3_ samples were 45.88, 45.78, 45.90 and 46.19 mg/100 g. High Performance Liquid Chromatography (HPLC) analysis showed that lactic acid, propionic acid, citric acid and acetic acid gradually and steadily increased during the storage interval of 90 days. At the age of 90 days, lactic acid in control, T_1_, T_2_ and T_3_ was 4,789, 5,487, 6,571 and 8,049 ppm, respectively. At the end of ripening duration of 90 days, free fatty acids in control, T_1_, T_2_ and T_3_ were 0.29, 0.31, 0.35 and 0.42% with no difference in peroxide value. Stability of vitamin A after 90 days storage control, T_1_, T_2_ and T_3_ was 87.0, 80.0, 94.0 and 91.0%. Flavor score of cheddar-type cheese produced from butter milk having 1.0, 2.5 and 3.25% fat content was 81, 89 and 91% of total score (9). Hence, it is concluded that cheddar-type cheese can be produced from buttermilk having 2.5 and 3.25% fat contents with acceptable sensory attributes. Application of buttermilk for the production of other cheese varieties should be studied.

## 1. Introduction

On the basis of acidity, two different types of buttermilk are produced, sweet buttermilk is produced during the churning of unfermented cream, cultured buttermilk is produced by fermentation of cream with mesophilic lactic acid producing bacteria (Walstra et al., 2005). Chemical composition of sweet butter is comprised of non-fat constituents of churned cream, i.e., casein, whey proteins, lactose and minerals, dry matter content of buttermilk varies from 8 to 12%. Fat globules in cream have an outer membrane. Milk fat globule membrane that covers the lipid core it provides stability and structural integrity to milk fat; this membrane is disrupted during churning then migrate to buttermilk. It helps fat globules in milk to stay in suspension because of its components that acts as emulsifier (phospholipids and glycoproteins). Magnitude of phospholipids in buttermilk ranges from 80 to 125 mg/g which is considerably higher than whole milk that contains 2.7–4.8 mg/g polar lipids (Sodini et al., 2006). Emulsifying, water holding and lower foaming capacity in buttermilk are due to phospholipids (Goudédranche et al., 2000). Biological properties of buttermilk such as antibacterial, cholesterol lowering, hypotensive, antioxidant potential, positive impact on nerve tissues, skin and hair are mainly due sphingomyelin, sialic acid and gangliosides (Walstra et al., 2005). High concentration of phospholipids in buttermilk are of great interest because that component possesses certain biological activities which have beneficial impact on human health. Studies indicate that phospholipids have potential to protect human body against bacterial toxins, infections and colon cancer. Along with this, it had emulsifying properties that is why this component had a significant importance as a functional ingredient that improves certain characteristics in food (Kifah et al., 2014).

Buttermilk is rich in lactic acid bacteria which boost immune system helps body to get rid of pathogens. Its probiotic effect lowers the risk of candida in diabetic women (Wang, 2019). A general analysis of buttermilk composition and chemical nature indicates that it has 0.18% acidity, 3.4% protein, 0.8% fat and 4% lactose reported by Shrestha et al. (2015).

One biggest challenge is to find ways for efficient utilization and applications of dairy by-products. One of the most significant by-products produced by the dairy industries is butter milk. During the churning process, considerable content of proteins and phospholipids are released from the membrane of fat globules to buttermilk that is perceived to have therapeutic effects (Sharma et al., 2021). In 2017, approximately 29% of the total milk produced in European Union was used for the production of butter, yield of butter and buttermilk was almost 50% (Eurostat, 2017). Due to its nutritional and technological properties, it is of great interest for the food industries to find/extend the applications of buttermilk for the development of functional foods. The possible application of buttermilk in fermented beverages and fruit drinks (Liutkevičius et al., 2016). However, cheese is supposed to be the biggest application area of buttermilk (Skryplonek et al., 2019).

Fat in cheese is extremely important for the functional characteristics and development of optimum textural attributes. Skryplonek et al. (2019) produced Quark cheese (un-ripened) from sweet and cultured buttermilks, composition, water activity, texture and sensory properties were studied however, fatty acids composition, lipolysis and sensorial prospects of ripened butter milk cheese is not reported in literature. Texture of cheddar-type cheese produced from buttermilk was soft with lack of flowability. Fat content in buttermilk-based cheddar-type cheese was about 10% (Hickey, 2017). Fatty acid composition of fat in buttermilk is considerably different from milk, former has a greater number of polyunsaturated fatty acids than the latter. Therefore, in this investigation, fat extracted from buttermilk was used as source of fat enrichment. Functional and textural properties of buttermilk-based cheddar type cheese can be improved by increasing fat content. Ripening of cheese has a great deal of impact on nutritional profile, texture and sensory properties of cheese. Low fat versions of cheese produced from buttermilk may be a good addition in cheese industry. Therefore, this investigation was conducted to know the effect of different fat contents of buttermilk on fatty acids composition, organic acids, vitamins, lipolysis and sensory characteristics of cheddar-type cheese.

## 2. Materials and methods

### 2.1. Starter culture

Cheddar cheese starter culture (CH-4219-CN) *Lactococcus lactis* ssp. lactis, *Lactococcus lactis* ssp. cremoris, Starter culture for butter milk (XT-313) *Lactococcus lactis* ssp. lactis biovar diacetylactis and *Leuconostoc mesenteroides* ssp. cremoris and rennet were obtained from Christian Hansen (Hoersholm, Denmark).

### 2.2. Buttermilk cheese production

Sweet buttermilk (having 1% fat content) was heated to 50°C in a water bath (Memmert water bath/Memmert GmbH & Co, KG) for the separation of cream from butter milk by cream separator (Packo, Belgium) separated cream was used to adjusted the fat content of butter milk to 1.75, 2.5 and 3.25% (T_1_, T_2_, T_3_ respectively). Buttermilk having 1% fat content was used as control (T_o_), pre-acidified for 45 min using 2% bulk starter culture of *Lactococcus lactis* spp. *lactis* and *Lactococcus lactis* spp. *cremoris*. Calcium chloride 2 mL (35%), rennet 0.02% were added and curd was cut to 1.5–2 cm cubes followed by cooking at 39°C for 45 min, cheddaring (pH 5.2), milling by cheddar cheese mill salting (1.5%), pressing by hydraulic press at 3.5 bar pressure for 16 h, vacuum packaging (by using a vacuum packaging machine/Tabletop vacuum – Sealing bar 265 mm) and ripening at 6–8°C for 90 days. Analysis was performed in triplicate on days 0, 45 and 90.

### 2.3. Composition of buttermilk and cheese

Compositional analysis such as fat, protein, lactose, ash, solids-not-fat, total solids content and pH of buttermilk and moisture, fat, protein and pH of cheddar-type cheese were performed as per standard procedures of AOAC (2005).

### 2.4. Fatty acid profile

First, extracted fat was mixed with methanolic hydrogen chloride (15 mL HCl and 85 mL methanol) in a screw capped test tube and was heated at 100°C for 60 min till fat was fully dissolved. After that, test tube was cooled down to room temperature. Further, 2 mL distilled water and 2 mL n hexane was added in the tubes followed by vortexing (Labnet’s Vortex Mixer VX-200) at 1,500 rpm for 2 min. Then the tube was placed down for 5 min or until two layers were separated. Then supernatant was poured in a tube containing 1 g anhydrous sodium sulphate, that were transferred to GC vials and injected to GC–MS, Agilent Technologies (7890-B), USA using a SP-2560 capillary column (75 m x 0.1 mm, DF 0.14 μm). With the split ratio of 50% injectors and 50% detectors were set at 250°C, using FAME-37 (Restek Corporation, USA) standards, flow rate of helium, hydrogen and oxygen were set at 2, 4 and 40 mL/min (Qian, 2003).

### 2.5. Organic acids by HPLC

Estimation of organic acids was performed on HPLC, 10 g sample with 40 mL dihydrogen phosphate (H_2_PO_4_) was taken in a tube and vortexed at 200 rpm for 1.5 min. Cheese samples were then incubated at 65°C and centrifugation (Beckman-S241.5) was performed at 2958 g for 5 min. By using Whatman filter paper the upper layer and lactic acid, propionic acid, citric acid and acetic acid were analyzed on a ShodexPSpak KC-118 (8 mm × 300 mm i.d.) ion exchange organic column attached with UV detector 214 nm column.

### 2.6. Lipolysis

As an indicator of lipolysis, free fatty acids (FFA), cholesterol and peroxide values (POV) were determined of all samples and control at 0, 45 and 90 days (American Oil Chemists’ Society, 2011). For measurement of FFA, sample (50 g) was mixed with absolute and neutralized ethanol with 0.1 N NaOH. Samples were then titrated with NaOH and FFA were calculated in terms of oleic acid. For POV, three parts of glacial acetic were blended with two parts of chloroform, 30 mL of this solution was mixed with samples, 1 g potassium iodide was added and flasks were incubated in dark for exactly 5 min and then titrated with standard 0.01 N sodium thiosulfate solution using starch (1% indicator) and POV was reported as (mEqO_2_/kg) and calculated with the help of following formula.


POV=(EQ1−B)∗T∗M∗F1/W∗F2

Where as:

B: Blank value; EQ: Consumption of titrant at first Equivalence point; T: Actual concentration of the titrant; M: Molecular weight; W: sample weight in g; F1: 1000 Conversion factor; F2: 1 Conversion factor.

### 2.7. Vitamin A determination by HPLC

A 20 mL buttermilk sample was taken and mixed with 5 mL of ammonia (25%) and 20 mL ethanol (96%). Supernatant was extracted and added with 0.0025% BHT and sol-vent was evaporated with the help of a rotary evaporator at 35°C. With the help of 30 mL potassium hydroxide (5% in ethanol) saponification was performed and extracted with n-hexane. After evaporation of solvent on rotary evaporator, 20 μL sample was injected into HPLC, Waters Corp., Milford, MA, USA/ (Shimadzu) which was equipped with Spherisorb RP-18 column, Supelcogel C-610H, 300 × 7.8 mm (Supelco Inc., Bellefonte, PA, USA) and water 990 detector. In various concentrations, retinyl palmitate was used as standard. Mobile phase was consisted of acetonitrile-methanol 85:15 (v/v) in isocratic system (Khan et al., 2017).

### 2.8. Vitamin E determination by HPLC

For vitamin E determination, fat was extracted by standard method. Then 2 mL *n*-hexane (HPLC grade) was added to 200 μg of fat. At 1,500 rpm the content was vortexed for 25 s and then injected (10 μL) into HPLC (HPLC LCM, Waters Corp., Milford, MA, USA/ (Shimadzu) which was equipped with Spherisorb RP-18 column, Supelcogel C-610H, 300 mm × 7.8 mm, Supelco Inc., Bellefonte, PA, USA). Mobile phase was comprised of 0.5% acetic acid and 0.5% ethyl acetate in n-hexane with a flow rate was adjusted at 1.5 mL/min. Vitamin E were expressed as μg/g (Khan et al., 2017).

### 2.9. Sensory evaluations

All the samples were tested for color, flavor and texture in individual sensory evaluation booths on 0, 45, and 90 days of storage. Ten trained judges performed the sensory evaluation using 9-point scale (Meilgaard et al., 2007).

### 2.10. Statistical analysis

The experiments were planned in a completely randomized design and data were analyzed using two-way analysis of variance. Duncan multiple range (DMR) tests were used to express significance difference different versions of cheddar-type cheese. DMR Test were used with the aid of SAS 9.4 statistical software (SAS Institute Inc. Cary, North Carolina, U.S.A).

## 3. Results and discussion

### 3.1. Cheese composition

Fat, protein, lactose, mineral, lactose and total solids contents in control milk were 1.00, 3.20, 2.95, 0.62 and 7.92%, respectively (Table 1). Difference in fat content of substrate buttermilk had a pronounced effect on fat and moisture content of cheddar-type cheese. It was recorded that conversion ratio of control and all samples were almost 10 times which is normal in cow milk cheddar cheese. From this conversion ratio, it is also perceived that this research work may be highly useful for the industries to develop functional buttermilk-based cheddar-type cheese. Further, cheddar-type cheese was produced from the same equipment which is used for the production of standard cheddar cheese and also standard cheese production procedure without any alteration was used. This will be helpful the industries to save time in the development and adoption of this cheese. Moisture content of control, T_1_, T_2_ and T_3_ were 62.52, 54.29, 45.67 and 40.35% (Table 1). Due to the presence of inverse relationship between moisture and fat, significant decline in moisture content of T_1_, T_2_ and T_3_ can be justified (Collins et al., 2003). Fat content of control, T_1_, T_2_ and T_3_ were 9.81, 16.34, 25.17 and 31.19%. Fat addition did not affect protein content and pH in control and experimental cheese samples. Fat level depends upon milk type, cheese manufacturing process and formulation. Fat level in cheese influences biochemistry, microstructure, yield and rheological properties of cheese (Murtaza et al., 2008). Cheddar-style cheese was produced either by blending buttermilk or buttermilk powder with cow milk, protein and fat content in cheese were 10 and 27%, respectively (Hickey et al., 2018). Ullah et al. (2018) reported that fat and protein content in standard cheddar cheese were 32 and 26%.

**Table 1:** Composition of cheddar-type cheese produced from buttermilk.

Treatment	Moisture (%)	Fat (%)	Protein (%)	pH
Control	62.52 ± 0.43^a^	9.81 ± 0.09^d^	24.11 ± 0.05^a^	5.21 ± 0.02^a^
T_1_	54.29 ± 0.37^b^	16.34 ± 0.15^c^	24.32 ± 0.08^a^	5.29 ± 0.05^a^
T_2_	45.67 ± 0.24^c^	25.17 ± 0.24^b^	24.61 ± 0.13^a^	5.24 ± 0.07^a^
T_3_	40.35 ± 0.64^d^	31.19 ± 0.13^a^	24.79 ± 0.17^a^	5.20 ± 0.10^a^

Control: Butter milk with 1% fat content. T_1_: Butter milk with 1.75% fat content. T_2_: Butter milk with 2.50% fat content. T_3_: Butter milk with 3.25% fat content. In a column, different letters on means indicate statistically significant difference (*p* < 0.05).

### 3.2. Fatty acids composition of cheddar type cheese

Among all dietary lipids, fatty acid composition of milk fat is highly complicated. Short-chain (C4:0-C10:0; SCFA), medium-chain (C12:0-C16:0; MCFA) and long chain (C18:0 and above LCFA) are present in milk fat. On an average, ratio of saturated and unsaturated fatty acids in milk is 70:30. Typical aroma, functional properties and nutritional characteristics of milk fat is due to mixture of fatty acids (Nadeem et al., 2014). Palmitic acid (C16:0) and stearic acid (C18:0) are the dominant fatty acids of milk. Processing technologies have a pronounced effect on fatty acid composition of fatty acid composition of milk. Fatty acid profile of butter and buttermilk were considerably different from each other, concentrations of oleic acid, linoleic acid and linolenic acid were significantly higher in buttermilk than butter and parent milk (Sakkas et al., 2022). Traditionally, cheddar cheese is ripened for several months to achieve optimum flavor and textural attributes. During this phase, lipolysis in milk fat takes place which leads to the production of free fatty acid (FFA), mono & di-glycerides, organic acids and flavoring compounds. In terms of auto-oxidative stability, cheese is relatively more resistant to lipid oxidation as compared to other dairy products and super oxidative stability of cheese is attributed to lower oxidation–reduction potential (McSweeney and Sousa, 2000). Ripening effect on fatty acid composition of standard cheese has been extensively studied however, transition in fatty acid composition of cheddar-type cheese produced exclusively from buttermilk is not reported in literature. In current investigation, GC–MS analysis showed that raising fat content of cheese buttermilk from 1% to 1.75, 2.5 and 3.25% did not affect fatty acids composition of cheddar type cheeses produced from buttermilk. This non-significant impact on fatty acid composition of control and experimental cheese samples was due to non-variation in source of fat, cream was extracted from buttermilk and fat contents of all the treatments were adjusted using same source of buttermilk cream. Determination of fatty acids composition in 90 days old cheese samples showed that ripening phase of 90 days had a significant influence on fatty acid composition of control, T_1_, T_2_ and T_3_. Cheddar-style cheese was produced either by blending milk with buttermilk or buttermilk powder, cheese samples were ripened for 180 days, it was found that fatty acids composition of 180 days old cheddar cheese was remarkably different from fresh cheese (Hickey, 2017). Most of lipolysis during the ripening phase of cheddar cheese is induced by starter bacteria. Khan et al. (2022) studied the impact of ripening on fatty acid composition of cheddar cheese, it was reported that ripening induced major changes in fatty acid composition of cheddar cheese. Batool et al. (2018a,b) monitored the effect of 42 days ripening period on fatty acid composition of cheddar cheese, fresh and 42 days old cheeses had different fatty acid composition (Ullah et al., 2018). Monitored ripening effect on fatty acid composition of cheddar cheese, freshly prepared curd and 3 months old cheddar cheese had considerably different fatty acid profile. In this investigation, two isomers of conjugated linoleic acids were also found, concentrations of C18:2c9t13 and C18:2c9c12 in control were 0.91 and 2.11 mg/100 g. Intake of 2 g conjugated linoleic acid on daily basis can prevent obesity, cancer, diabetes and cardiovascular diseases (Khan et al., 2021; Table 2).

**Table 2:** Fatty acids composition of cheddar-type cheese produced from buttermilk (mg/100 g).

Fatty Acid	Control		T_1_		T_2_		T_3_	
	0-Day	90-Days	0-Day	90-Days	0-Day	90-Days	0-Day	90-Days
C4:0	1.81 ± 0.01^a^	1.62 ± 0.05^b^	1.80 ± 0.03^a^	1.61 ± 0.08^b^	1.79 ± 0.04^a^	1.59 ± 0.07^b^	1.78 ± 0.02^a^	1.48 ± 0.10^c^
C6:0	2.15 ± 0.02^a^	1.94 ± 0.03^b^	2.17 ± 0.06^a^	1.91 ± 0.05^b^	2.15 ± 0.07^a^	1.90 ± 0.02^b^	2.14 ± 0.05^a^	2.05 ± 0.03^b^
C8:0	2.49 ± 0.05^a^	2.27 ± 0.01^b^	2.51 ± 0.01^a^	2.25 ± 0.07^b^	2.50 ± 0.01^a^	2.23 ± 0.09^b^	2.48 ± 0.08^a^	2.21 ± 0.05^b^
C10:0	3.25 ± 0.10^a^	2.88 ± 0.19^b^	3.26 ± 0.04^a^	2.86 ± 0.09^b^	3.25 ± 0.09^a^	2.85 ± 0.04^b^	3.24 ± 0.16^a^	2.84 ± 0.02^b^
C12:0	2.77 ± 0.11^a^	2.53 ± 0.13^b^	2.75 ± 0.07^a^	2.49 ± 0.13^b^	2.73 ± 0.03^a^	2.46 ± 0.17^b^	2.71 ± 0.04^a^	2.68 ± 0.12^b^
C14:0	10.84 ± 0.19^a^	9.24 ± 0.26^b^	10.88 ± 0.32^a^	9.14 ± 0.18^b^	10.79 ± 0.27^a^	10.99 ± 0.13^b^	10.85 ± 0.24^a^	10.74 ± 0.34^b^
C16:0	17.69 ± 0.24^a^	16.21 ± 0.39^b^	17.95 ± 0.46^a^	16.10 ± 0.34^b^	17.82 ± 0.28^a^	17.93 ± 0.34^b^	17.88 ± 0.41^a^	17.16 ± 0.21^b^
C18:0	8.77 ± 0.13^a^	7.44 ± 0.17^b^	8.82 ± 0.21^a^	7.35 ± 0.38^b^	8.77 ± 0.42^a^	8.83 ± 0.19^b^	8.91 ± 0.35^a^	7.82 ± 0.07^b^
C18:1	36.43 ± 0.17^a^	34.59 ± 0.48^b^	36.55 ± 0.54^a^	34.49 ± 0.66^b^	36.42 ± 0.77^a^	36.79 ± 0.90^b^	36.67 ± 0.83^a^	35.87 ± 0.25^b^
C18:2	7.66 ± 0.09^a^	6.89 ± 0.08^b^	7.59 ± 0.12^a^	6.81 ± 0.02^b^	7.78 ± 0.15^a^	7.91 ± 0.56^b^	7.74 ± 0.27^a^	7.13 ± 0.19^b^
C18:3	1.79 ± 0.06^a^	1.63 ± 0.02^b^	1.74 ± 0.05^a^	1.61 ± 0.15^b^	1.70 ± 0.04^a^	1.69 ± 0.47^b^	1.78 ± 0.04^a^	1.44 ± 0.14^b^
C18:2*c*9*t*13	0.91 ± 0.04^a^	0.75 ± 0.01^b^	0.89 ± 0.03^a^	0.80 ± 0.03^b^	0.88 ± 0.02^a^	0.82 ± 0.06^b^	0.91 ± 0.03^a^	0.81 ± 0.02^b^
C18:2*c*9*c*12	2.11 ± 0.03^a^	1.94 ± 0.03^b^	2.15 ± 0.01^a^	1.91 ± 0.02^b^	2.10 ± 0.01^a^	2.05 ± 0.11^b^	2.13 ± 0.02^a^	2.06 ± 0.01^b^

Control: Butter milk with 1% fat content. T_1_: Butter milk with 1.75% fat content. T_2_: Butter milk with 2.50% fat content. T_3_: Butter milk with 3.25% fat content. In a row, different letters on means indicate statistically significant difference (*p* < 0.05).

### 3.3. Organic acids

In several ripened cheese varieties, organic acids are an important flavoring compounds, which are produced due to the breakdown milk fat, addition of acids, usual ruminant metabolic courses or the by the growth of bacteria. Organic acids are also produced by the catabolism of carbohydrates by the activities of lactic acid bacteria (Ullah et al., 2018). Organic acids can exert a great deal of control on undesirable bacteria by dropping pH but inhibitory effect largely depends upon the kinds of organic acids produced. Extent of lipolysis varies among different varieties of cheese, in cheddar, Gouda and Emmentaler cheese, medium degree of lipolysis is required however, in certain hard Italian cheese varieties, and extensive lipolysis is required for the development of desired flavor (Murtaza et al., 2014). Fat segment of cheese is extremely important for the production of typical flavor and texture cheese. Standard cheddar cheese contains more than 30% fat, in addition to other factors, degree of lipolysis is also largely determined by the fat content of cheese. Low fat versions of cheddar cheese undergo lower degree of lipolysis than standard cheddar cheese (McSweeney and Sousa, 2000). In current investigation, impact of different fat levels, i.e., 1, 1.75, 2.5 and 3.25% in cheese buttermilk and ripening period of 90 days was investigated on production of organic acids. Results showed that fat contents had a great deal of impact on generation of organic acids, highest organic acids production was recorded in T_3_ followed by T_2_, T_1_ and control (*p* < 0.05). At the age of 45 days, lactic acid in control, T_1_, T_2_ and T_3_ was 4,571, 4,829, 5,689 and 7,019 ppm (Table 3). At the age of 45 days, citric acid in control, T_1_, T_2_ and T_3_ was 18,743, 20,443, 26,213 and 36,641 ppm. Concentrations of lactic acid, propionic acid, citric acid and acetic gradually and steadily increased during the storage interval of 90 days. At the age of 90 days, lactic acid in control, T_1_, T_2_ and T_3_ was 4,789. 5,487. 6,571 and 8,049 ppm. At the age of 90 days, citric acid in control, T_1_, T_2_ and T_3_ was 20,465, 24,519, 33,101 and 47,993 ppm. Conventionally, cheddar cheese is produced from cow milk with a fat content of 3.2 to 3.5%, for the production of cheddar cheese from buffalo milk (4% fat content) was used, impact of higher fat content in cheese milk was studied on production of organic acids. Concentrations of organic acids were higher when high fat cheese milk was used to produce cheddar cheese as compared to standard cheese (Murtaza et al., 2012; Ikram et al., 2021).

**Table 3:** Organic acids of cheddar-type cheese produced from buttermilk (ppm).

Cheese type	Days	Lactic acid	Propionic acid	Citric acid	Acetic acid
Control	45	4,571 ± 1.24^h^	217 ± 0.03^g^	108 ± 0.05^h^	18,743 ± 2.56^h^
	90	4,789 ± 2.57^g^	342 ± 0.09^f^	243 ± 0.03^g^	20,465 ± 3.17^g^
T_1_	45	4,829 ± 3.49^f^	379 ± 0.81^e^	243 ± 0.15^f^	20,443 ± 5.77^f^
	90	5,487 ± 1.98^e^	614 ± 0.21^d^	379 ± 0.13^e^	24,519 ± 0.98^e^
T_2_	45	5,689 ± 2.19^d^	527 ± 0.36^e^	464 ± 0.24^d^	26,213 ± 0.73^d^
	90	6,571 ± 1.77^c^	884 ± 0.28^c^	713 ± 0.17^c^	33,101 ± 1.35^c^
T_3_	45	7,019 ± 1.35^b^	1,034 ± 0.15^b^	984 ± 0.29^b^	34,641 ± 1.61^b^
	90	8,049 ± 0.65^a^	1,437 ± 0.44^a^	1,481 ± 0.35^a^	47,993 ± 1.43^a^

Control: Butter milk with 1% fat content. T_1_: Butter milk with 1.75% fat content. T_2_: Butter milk with 2.50% fat content. T_3_: Butter milk with 3.25% fat content. In a column, different letters on means indicate statistically significant difference (*p* < 0.05).

### 3.4. Lipolysis

Due to action of lipases, enzymatic hydrolysis takes place during the ripening of cheese that improve flavor of cheese by producing FFA which are further catabolized to flavoring compounds (McSweeney and Sousa, 2000). Proportions of fatty acids from C6:0 to C18:3 is almost similar to milk fat which shows that hydrolysis of triglycerides and production of FFA is non-specific. Degree of lipolysis varies from cheese to cheese, in several varieties of cheese, lower level of lipolysis of desirable for optimum flavor and too much lipolysis is undesirable. In cheddar, Gouda and Swiss cheeses, moderate levels of free acids are required. However, extensive lipolysis occurs during the ripening of certain hard Italian, Blue and Feta cheese. Lipolysis is affected by moisture, temperature, ripening duration, fatty acid composition, fat content and oxygen, methyl ketones, thioesters and lactones are produced due to the catabolism of FFA. Oxidation of fatty acid result in the production of ketoacids which are carboxylated to methyl ketones mainly from capric acid to lauric acid (Alewijn et al., 2005). Effect of different contents on lipolysis of cheddar type-cheese is documented in Table 4. Transition in values of FFA, POV and cholesterol were used to estimate the degree of lipolysis. Before ripening, FFA of control, T_1_, T_2_ and T_3_ were similar to each other (*p* > 0.05). FFA of buttermilk cream were 0.08% and same source of cream was used in T_1_, T_2_ and T_3_ levels. Estimation of FFA in 45 days old cheddar-type cheese samples revealed a significant impact on ripening on production of ripening. Treatments having higher fat content yielded more FFA and were in the order of T_3_ > T_2_ > T_1_ > control (Table 4). At the end of ripening duration of 90 days, FFA in control, T_1_, T_2_ and T_3_ were 0.29, 0.31, 0.35 and 0.42%. Butter oil was added to cheese milk and converted to cheese, cheese having buttermilk produced more FFA as compared to cheese prepared without addition of butter oil (Morin et al., 2007). Ahmad et al. (2015) monitored the generation of FFA in cheese during the time frame of 3 months, it was observed that FFA in cheddar cheese slowly steadily increased in the duration of 3 months. Magnitudes of unsaturated fatty acids is normally higher in buttermilk than milk, the resilience of unsaturated fatty acids such as linoleic acid is hundred times lesser than stearic acid. Therefore, control over lipid oxidation in buttermilk-based cheese and other products is challenging for the food scientists. In present study, cheddar-type cheese was produced from buttermilk having four different levels of fat and POV was used to measure lipid oxidation at regular frequencies of 0, 45 and 90 days. Different fat contents in control, T_1_, T_2_ and T_3_ did not affect POV however, POV constantly increased in the ripening phase of cheddar-type cheese. The rise in POV of control, T_1_, T_2_ and T_3_ during the entire ripening phase was not due to the existence of a greater number of unsaturated fatty acids in buttermilk than standard milk and different fat contents in treatments but it was due to the ripening effect. According to the guidelines of European Union, maximum allowable limit of FFA and POV is 0.2% and 10 (MeqO_2_/kg). At the end of ripening, POV of control was 0.68 (mEqO2/kg) which was almost similar to cow cheddar cheese (Batool et al., 2018a,b). Due to lipid oxidation in food matrix, objectionable flavors may be developed, ripened cheeses usually do not suffer from lipid oxidation because of lower oxidation reduction potential. Addition of buttermilk in several foods inhibited lipid oxidation (Vanderghem et al., 2010). Among all the treatments and control cholesterol significantly varied when analyzed at 0, 45 and 90 days of ripening. Significant variation in cholesterol content was due to the presence of higher fat content in T_1_, T_2_ and T_3_. Ripening period of 90 days remarkably reduce cholesterol in control, T_1_, T_2_ and T_3_. Khan et al. (2018) monitored the changes in concentration of cholesterol during the ripening duration of 90 days in Gouda cheese, results showed that cholesterol content of 90 days old cheese were lower than fresh cheese.

**Table 4:** Lipolysis of cheddar-type cheese produced from buttermilk.

Cheese type	Days	Free fatty acids (%)	Peroxide value (mEqO_2_/kg)	Cholesterol (mg/dL)
Control	0	0.08 ± 0.01^g^	0.25 ± 0.02^c^	112.29 ± 0.68^e^
	45	0.16 ± 0.02^f^	0.42 ± 0.05^b^	103.27 ± 0.07^f^
	90	0.29 ± 0.01^c^	0.68 ± 0.03^a^	89.24 ± 0.09^g^
T_1_	0	0.08 ± 0.01^g^	0.25 ± 0.02^c^	132.47 ± 0.51^c^
	45	0.19 ± 0.03^e^	0.39 ± 0.07^b^	121.49d ± 0.14^d^
	90	0.31 ± 0.02^c^	0.65 ± 0.04^a^	108.72 ± 0.26^e^
T_2_	0	0.08 ± 0.01^g^	0.25 ± 0.02^c^	157.58 ± 0.13^b^
	45	0.22 ± 0.02^d^	0.44 ± 0.01^b^	142.33 ± 0.25^b^
	90	0.35 ± 0.01^b^	0.61 ± 0.03^a^	125.79 ± 0.18^d^
T_3_	0	0.08 ± 0.02^g^	0.25 ± 0.02^c^	177.83 ± 0.91^a^
	45	0.27 ± 0.04^c^	0.40 ± 0.07^b^	161.27 ± 0.19^b^
	90	0.42 ± 0.03^a^	0.71 ± 0.06^a^	139.89 ± 0.12^c^

Control: Butter milk with 1% fat content. T_1_: Butter milk with 1.75% fat content. T_2_: Butter milk with 2.50% fat content. T_3_: Butter milk with 3.25% fat content. In a column, different letters on means indicate statistically significant difference (*p* < 0.05).

### 3.5. Vitamin A and E

Milk and dairy products are fundamental part of human diet and their intake may from 5 to 10%. Increased awareness metabolic diseases, nutrition, transition in lifestyles and availability of wide range of functional foods has reduced in the consumption of dairy products all over the word. Food scientists and technologists have not only developed new but also converted the traditional foods to functional foods (Khan et al., 2022). In this study, an effort was made to develop cheddar-type cheese using buttermilk having different fat contents as a substrate as it is a good source of vitamin A and E. Vitamins belong to the group of organic compounds and perform several physiologically important functions in human body. Vitamin A is required for good vision, it helps the eyes to adjust in diffused light, as a catalyst of proteins and part of several enzyme systems. Vitamin E possess antioxidant properties and it can help the body to slower down ageing process. About 10% of the daily requirements of vitamin A is obtained from dairy products (Górska-Warsewicz et al., 2019). Batool et al. (2018a,b) studied the impact of vitamin E addition in cheddar cheese, results showed that vitamin E raised antioxidant capacity and stability with no effect on sensory properties. Neutralization of free radicals is mandatory to prevent oxidative stress that can cause cancer, diabetes, atherogenesis, lipid and protein oxidation. Effect of different contents and ripening on vitamin A and E in cheddar-type cheese was analyzed and documented (Table 5). In fresh cheese, vitamin A and E contents were significantly higher in experimental cheese samples than the control and were in the order of T_3_ > T_2_ > T_1_ > control. Stability of vitamin A in 90 days old control, T_1_, T_2_ and T_3_ was 87, 80, 94 and 91%, respectively. Stability of vitamin E in 90 days old control, T_1_, T_2_ and T_3_ was 82, 87, 86 and 91%, respectively (Ikram et al., 2021). Supplemented cheddar cheese with vitamin A four different concentrations, i.e., 3,500, 4,000, 4,500 and 5,000 IU/kg and stability was analyzed till 3 months in refrigerated stored cheese. In all treatments and control, stability of vitamin A was more than 80%. Stability of vitamin in buttermilk, Gouda cheese, yoghurt and pasteurized milk was more than 80% (Park, 2009). Microcapsules of vitamin A and E were added to cheddar cheese, in mature cheddar cheese, stability of vitamin A and E was 90 and 82% (Stratulat et al., 2014). Stability of vitamin A and in buttermilk derived cheese was greater than 90% (Król et al., 2020).

**Table 5:** Vitamin A and E in cheddar-type cheese produced from buttermilk.

Treatment	Days	Vitamin A (IU/100 g)	Vitamin E (mg/100 g)
Control	0	360.57 ± 0.31^g^	1.16 ± 0.07^g^
	90	314.29 ± 0.41^h^	0.95 ± 0.04^h^
T_1_	0	570.14 ± 0.27^e^	1.72 ± 0.02^e^
	90	456.38 ± 0.25^f^	1.49 ± 0.08^f^
T_2_	0	834.67 ± 0.39^c^	1.89 ± 0.04^c^
	90	791.18 ± 0.54^d^	1.63 ± 0.06^d^
T_3_	0	1136.57 ± 0.44^a^	2.31 ± 0.09^a^
	90	1039.47 ± 0.89^b^	2.10 ± 0.03^b^

Control: Butter milk with 1% fat content. T_1_: Butter milk with 1.75% fat content. T_2_: Butter milk with 2.50% fat content. T_3_: Butter milk with 3.25% fat content. In a column, different letters on means indicate statistically significant difference (*p* < 0.05).

### 3.6. Sensory characteristics

Fat content significantly affected the flavor and texture score of cheddar-type cheese produced from buttermilk having 1, 1.75, 2.5 and 3.25% fat. Flavor score of 45 days old control, T_2_ and T_3_ were at par with each other (*p* < 0.05). Flavor score of all the treatments was strongly correlated with POV, non-variation in flavor score of control, T_2_ and T_3_ was due to minimum variation in peroxide value. Flavor score and POV of 90 days old cheddar cheese and control were strongly correlated (Batool et al., 2018a,b). Ullah et al. (2018) reported that fat content had a significant effect on flavor and texture of cheddar cheese. Texture score of 45 days old control, T_2_ and T_3_ were 7.0, 8.0 and 8.1 (*p* < 0.05). Ripening of 90 days considerably improved flavor and texture score, highest flavor score was obtained by T_3_ followed by T_2_. After 90 days, highest texture score was obtained by T_3_ followed by T_2_. Texture scores of T_1_ and control were not different from each other. Flavor score of cheddar-type cheese produced from butter milk having 1, 2.5 and 3.25% fat content was 81, 89 and 91% of total score (9). These results suggested that cheddar-type cheese can be produced from buttermilk having 2.5 and 3.25% fat contents with acceptable sensory attributes (Table 6).

**Table 6:** Sensory properties of cheddar-type cheese produced from buttermilk.

Cheese type	Days	Color	Flavor	Texture
Control	45	8.1 ± 0.14^a^	7.2 ± 0.13^c^	7.0 ± 0.04^c^
	90	8.3 ± 0.11^a^	7.3 ± 0.18^b^	7.4 ± 0.02^b^
T_1_	45	8.2 ± 0.06^a^	7.3 ± 0.23^c^	7.1 ± 0.09^c^
	90	8.4 ± 0.19^a^	7.6 ± 0.35^b^	7.3 ± 0.03^b^
T_2_	45	8.2 ± 0.04^a^	7.9 ± 0.11^a^	8.0 ± 0.17^a^
	90	8.3 ± 0.08^a^	8.0 ± 0.28^a^	8.2 ± 0.28^a^
T_3_	45	8.1 ± 0.05^a^	8.0 ± 0.05^a^	8.1 ± 0.02^a^
	90	8.3 ± 0.13^a^	8.2 ± 0.26^a^	8.3 ± 0.06^a^

Control: Butter milk with 1% fat content. T_1_: Butter milk with 1.75% fat content. T_2_: Butter milk with 2.50% fat content. T_3_: Butter milk with 3.25% fat content. In a column, different letters on means indicate statistically significant difference (*p* < 0.05).

## 4. Conclusion

The aim of this study was to estimate the impact of buttermilk and their cream on fatty acid composition, organic acid, oxidative stability and vitamins contents, as well as sensory properties of experimental cheddar-style cheese samples. The results of samples showed a non-significant effect on fatty acid composition in comparison to the control. In contrast, organic acids in the experimental cheese samples were significantly improved at the end of the 90-day ripening period. Free fatty acid was slightly increased in the cheese sample, while peroxide value of cheddar-style cheese samples resulted in similar values compared to the control. Furthermore, vitamins A and E was stable in the experimental cheese samples during the storage period, and sensory scores for color, taste, texture were within acceptable range. This study may open a new array for emerging innovative product in the healthy cheese market, with relevant potential of nutritional properties. Further research work could be done on the use of buttermilk cream formulation as a method of improving bioactive properties of cheddar-style cheese in the diet.

## Data availability statement

The original contributions presented in the study are included in the article/supplementary material, further inquiries can be directed to the corresponding authors.

## Author contributions

MA performed the methods and investigation. MN was involved in conceptualization, funding acquisition, and writing of original draft. MI helped in writing of this manuscript. RU, FA-A, and MT helped in software. JMR, SK, FK, MAR, and TE supported in analysis and supervision of research work. TE supported in funding. All authors contributed to the article and approved the submitted version.

## Funding

Funding for this study was provided by Higher Education Commission of Pakistan (HEC) under National Research Program for Universities (NRPU/6978). This work was also supported by the Deanship of Scientific Research, Vice Presidency for Graduate Studies and Scientific Research, King Faisal University, Saudi Arabia [Grant no. 3923]. The publication of this article was funded by the Open Access Fund of Leibniz Universität Hannover.

## Conflict of interest

The authors declare that the research was conducted in the absence of any commercial or financial relationships that could be construed as a potential conflict of interest.

## Publisher’s note

All claims expressed in this article are solely those of the authors and do not necessarily represent those of their affiliated organizations, or those of the publisher, the editors and the reviewers. Any product that may be evaluated in this article, or claim that may be made by its manufacturer, is not guaranteed or endorsed by the publisher.
